# Identifying circRNA-associated-ceRNA networks in retinal neovascularization in mice

**DOI:** 10.7150/ijms.35149

**Published:** 2019-09-20

**Authors:** Manjing Cao, Lusi Zhang, Jiang-Hui Wang, Huilan Zeng, Yingqian Peng, Jingling Zou, Jingming Shi, Liwei Zhang, Yun Li, Shigeo Yoshida, Luosheng Tang, Yedi Zhou

**Affiliations:** 1 Department of Ophthalmology, The Second Xiangya Hospital, Central South University, Changsha, Hunan 410011, China; 2 Hunan Clinical Research Center of Ophthalmic Disease, Changsha, Hunan 410011, China; 3 Centre for Eye Research Australia, Royal Victorian Eye and Ear Hospital, East Melbourne, Victoria, Australia; 4 Ophthalmology, Department of Surgery, University of Melbourne, East Melbourne, Victoria, Australia; 5 Department of Ophthalmology, Kurume University School of Medicine, Kurume, Fukuoka 830-0011, Japan

**Keywords:** circular RNA (circRNA), expression profile, microarray, oxygen-induced retinopathy, retinal neovascularization

## Abstract

Retinal neovascularization is a complication which caused human vision loss severely. It has been shown that circular RNAs (circRNAs) play essential roles in gene regulation. However, circRNA expression profile and the underlying mechanisms in retinal neovascular diseases remain unclear. In the present study, we identified altered circRNAs in the retinas of oxygen-induced retinopathy (OIR) mouse model by microarray profiling. Microarray analysis revealed that 539 circRNAs were significantly altered in OIR retinas compared with controls. Among them, 185 up-regulated and 354 down-regulated circRNAs were identified. The expression levels of 4 altered circRNAs including mmu_circRNA_002573, mmu_circRNA_011180, mmu_circRNA_016108 and mmu_circRNA_22546 were validated by quantitative real-time reverse transcription-polymerase chain reaction (qRT-PCR). Bioinformatic analysis with validated circRNAs such as competing endogenous RNA (ceRNA) regulatory networks with Gene Ontology (GO) enrichment analysis demonstrated that qRT-PCR validated circRNAs were associated with cellular process, cell part and phosphoric ester hydrolase activity. Kyoto Encyclopedia of Genes and Genomes (KEGG) pathway analysis demonstrated that MAPK signaling pathway and renin-angiotensin system were related to validated circRNAs, suggesting these pathways may participate in pathological angiogenesis. The results together suggested that circRNAs were aberrantly expressed in OIR retinas and may play potential roles in retinal neovascular diseases.

## Introduction

Ischemia-induced retinal neovascularization is a key pathological process of many retinal vascular diseases like diabetic retinopathy and retinopathy of prematurity (ROP). It is also a leading cause of vision loss as well as blindness in developed countries and regions 1. Studies have shown that a variety of altered genes are involved in the pathogenesis of retinal neovascularization 2, 3. Several molecules, such as vascular endothelial growth factor (VEGF), basic fibroblast growth factor as well as periostin, have been identified as key mediators in pathological angiogenesis 4, 5. In particular, VEGF has been widely studied and targeted in clinical applications where reports showed anti-VEGF treatment was remarkably efficient in patients 6. However, some patients are not responsive to anti-VEGF therapy 7, suggesting further studies should aim to look for other therapeutic targets and biomarkers.

Circular RNA (circRNA), as a type of endogenous non-coding RNA, is characterized by covalently closed continuous loop structure lacking 5' cap and poly(A) tail at 3' ends 8. CircRNA is widely expressed in serum and tumor tissues in mammals 9, 10. CircRNA functions as a molecular sponge efficiently targeting miRNA and inhibiting miRNA transcription 11, 12. Through targeting miRNA, circRNA regulates downstream gene expression and may play a crucial role in disease mechanisms 13. A few studies have demonstrated that several circRNAs participate in the pathogenesis of diabetic retinopathy 14, 15, vascular endothelial cells proliferation and angiogenesis 16, suggesting circRNAs could serve as potential biomarkers for diagnosis of diabetic retinopathy and provide novel therapeutic targets to treat diabetic retinopathy. A study reported that circRNA-MYLK served as competing endogenous RNA (ceRNA) for miR-29a, contributing to epithelial-mesenchymal transition and the bladder cancer progression by activating VEGFA/VEGFR2 pathway 17. Besides, circRNAs ZNF280C_hsa_circ_001211 and SIAE_ hsa_circ_002083 may participate in the key pathways of ROP pathogenesis 18. However, the involvement of other potential circRNAs and the underlying mechanisms of ROP remain unclear.

We previously demonstrated differentially expressed mRNAs and long non-coding RNAs in the oxygen-induced retinopathy (OIR) mouse model 19, suggesting that non-coding RNAs played crucial and different roles in retinal neovascularization. In this study, we performed microarray analysis aiming to profile expressions of another kind of emerging non-coding RNA, circRNA in retinas of OIR mice. Furthermore, we interrogated the putative functions of the altered circRNAs by bioinformatic analysis.

## Materials and Methods

### Animals and statement of ethics

C57BL/6J mice (Hunan SJA Laboratory Animal, Changsha, China) were used in the present study. The animal experiments were conducted based on ARVO Statement for the Use of Animals in Ophthalmic and Vision Research, and were subjected to approval by the Institutional Animal Care and Use Committee of Central South University.

### Oxygen-induced retinopathy mouse model

The OIR mouse model was established as described 20. Pups were exposed to hyperoxia environment (75% oxygen) for 5 days at postnatal day 7 (P7), followed by return to the environment of room air at P12. Newborn pups kept continuously in the room air environment were used as the controls. The retina samples were collected from mice of both groups at P17 for analysis.

### RNA isolation

Total RNAs were isolated from retinas (retinas from both eyes of the same mice were pooled as one sample) by using TRIzol reagent (Invitrogen, Carlsbad, USA). The concentrations were assessed by the NanoDrop ND-1000 (Thermo Scientific, Wilmington, DE, USA). The RNA integrity was examined by electrophoresis on a denaturing agarose gel prior to further investigation.

### Microarray analysis of circRNAs

Microarray analysis is widely used to identify altered circRNAs in *in vivo* studies 21. Three pairs of retinas in each group were used for the analysis. Sample labeling and microarray hybridization were conducted according to the standard protocols Arraystar (Rockville, MD, USA). Briefly, total RNAs were digested with Rnase R (Epicentre, Madison, WI, USA). A random priming method with the Arraystar Super RNA Labeling Kit was utilized to amplify the enriched circRNAs and to transcribe the circRNAs into fluorescent cRNA. Labeled cRNAs were hybridized onto Mouse circRNA Array V2 (8x15K, Arraystar). Then the slides were washed, and the arrays were then scanned by Agilent Scanner G2505C, and the images were analyzed by an Agilent Feature Extraction software (version 11.0.1.1). Detected circRNAs were regarded as significantly differentially expressed by the value of fold change≥1.5 and *P*<0.05.

### Validation of quantitative real-time reverse transcription-polymerase chain reaction

To validate the data of microarray analysis, quantitative real-time reverse transcription-polymerase chain reaction (qRT-PCR) was performed as previously described 22. Briefly, total RNAs were transcribed into cDNAs by the SuperScript III Reverse Transcriptase kit (Invitrogen, Carlsbad, CA, USA). The ViiA 7 Real-Time PCR System (Applied Biosystems, Foster City, CA, USA) as well as 2× PCR Master Mix were used to perform the qRT-PCR. The primers used for qRT-PCR were listed in Table 1. The relative expression levels of circRNAs were normalized to GAPDH.

### Bioinformatics analysis

According to TargetScan (http://www.targetscan.org) and miRanda (http://www.microrna.org), the miRNA target prediction software (Arraystar) was used to predict circRNA-miRNA interactions. Cytoscape was used to predict a circRNA-miRNA-mRNA network to further visualize the interactions. To forecast the functional annotation of target genes, Gene Ontology (GO) analysis and Kyoto Encyclopedia of Genes and Genomes (KEGG) pathway analysis were used for further bioinformatics analysis.

### Statistical Analyses

In the present study, statistical differences were assessed by *Student t*-test, and *P*<0.05 was considered as statistically significant.

## Results

### Expression profiling of altered circRNAs in OIR retinas

**Figure 1:**
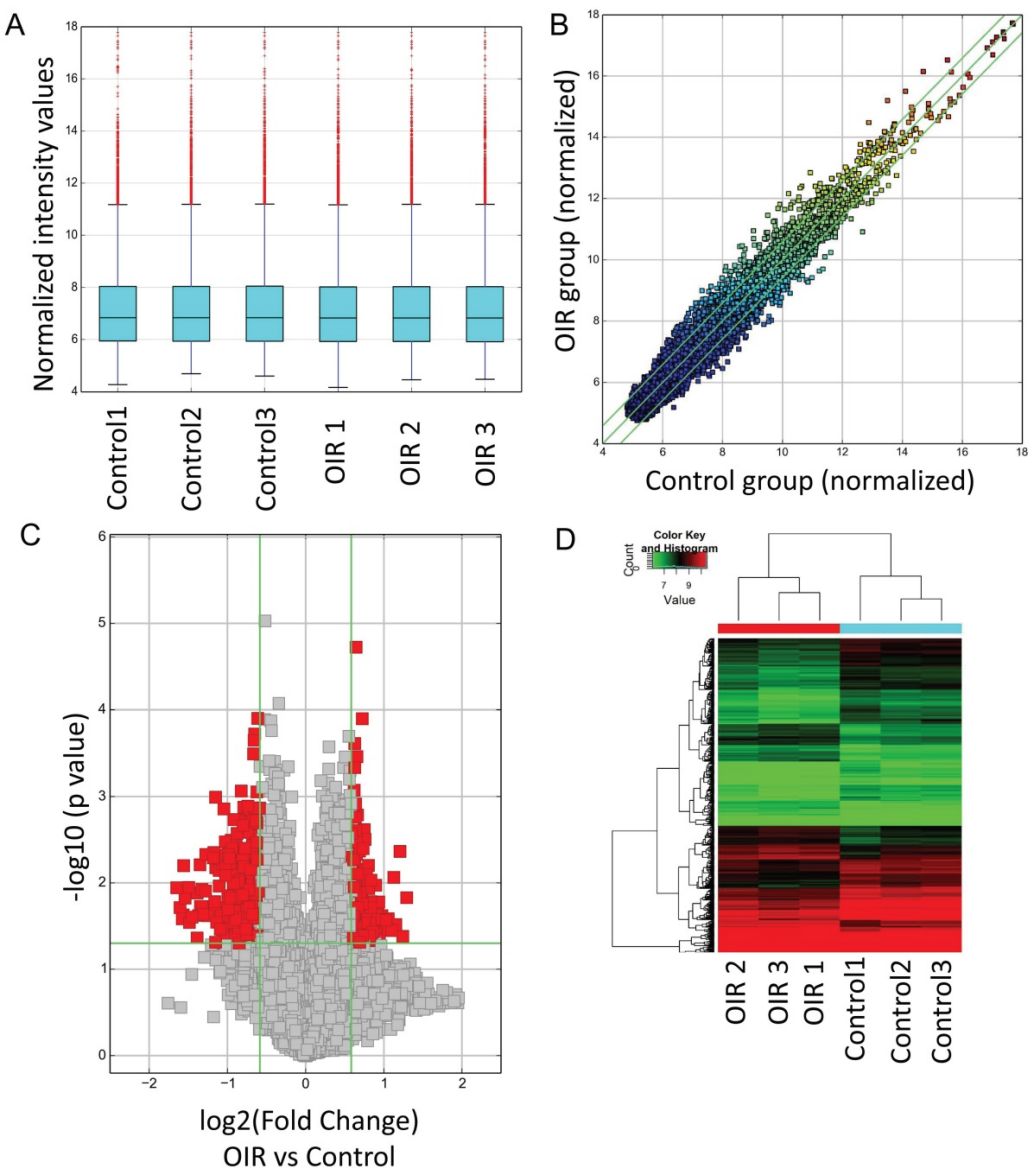
** Altered circRNAs between OIR retinas and room air control retinas by microarray analysis. A.** Box plot. The expression profiles of circRNAs were shown as a box plot after normalization. **B.** Scatter plot. A scatter plot is used to show the raw variations of the expression profile between the two groups. **C**. Volcano plot. The horizontal line represents P=0.05, and the red points represent the statistical altered circRNAs. The green lines in B and C represent the default significant fold change (1.5). **D.** Hierarchical cluster analysis (heat map) represents all of the altered circRNAs between the two groups. Red and green denoted high and low expression, respectively.

CircRNA expressions between OIR retinas and room air control retinas were measured by microarray. The analysis of hierarchical cluster identified different circRNA expression levels in OIR group and control group. A box plot showing the circRNA profiles (Fig. 1A) indicated similar distributions of all included samples in both groups. The variation of these detected circRNAs between each group was assessed by the scatter plot (Fig. 1B) and the volcano plot (Fig. 1C). In addition, the hierarchical cluster analysis showed the altered circRNA expression levels as well as the classifications in OIR group and control group. Totally 539 circRNAs were significantly altered in OIR retinas compared with control retinas (fold change≥1.5, *P*<0.05). Of which, 185 circRNAs were significantly up-regulated, and 354 circRNAs were significantly down-regulated in the OIR retinas (Fig. 1D). The top 20 significantly up- and down-regulated circRNAs were listed in Table 2-3. In particular, mmu_circRNA_007438 and mmu_circRNA_012434 were up/down-regulated circRNAs with the most significant alterations in OIR retinas.

### Validation of altered circRNAs by qRT-PCR

**Figure 2:**
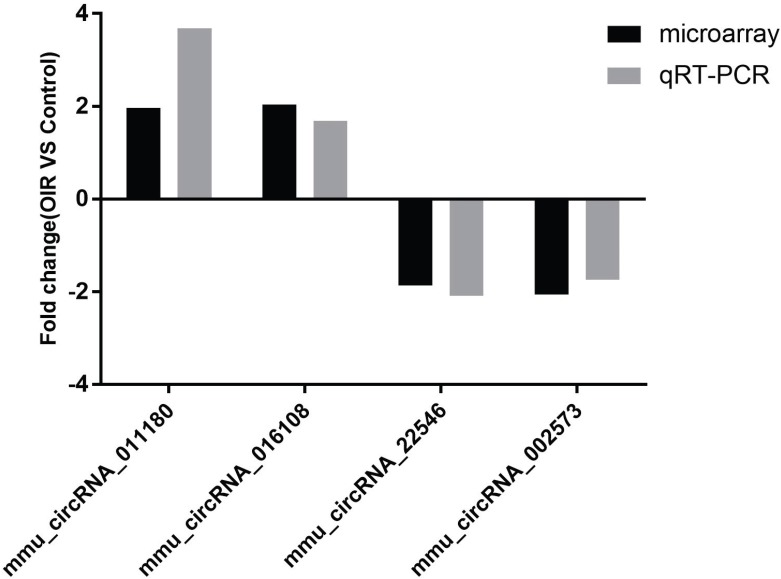
The relative expressions of candidate circRNAs in OIR group and control group for validation assessed by qRT-PCR.

Four of the significantly altered circRNAs including mmu_circRNA_002573, mmu_circRNA_011180, mmu_circRNA_016108 and mmu_circRNA_22546 were validated by qRT-PCR (Fig. 2). The results showed that the expressions of mmu_circRNA_011180 and mmu_circRNA_016108 were significantly increased in OIR retinas (3.68-fold, *P*<0.01 and 1.69-fold, *P*<0.05, respectively). Likewise, mmu_circRNA_002573 and mmu_circRNA_22546 were significantly decreased in OIR retinas (1.74-fold, *P*<0.05 and 2.10-fold, *P*<0.05, respectively). The qRT-PCR showed similar trend with microarray analysis, suggesting the reliability of our circRNAs expression profile by microarray.

### Prediction of circRNA-miRNA interactions

**Figure 3:**
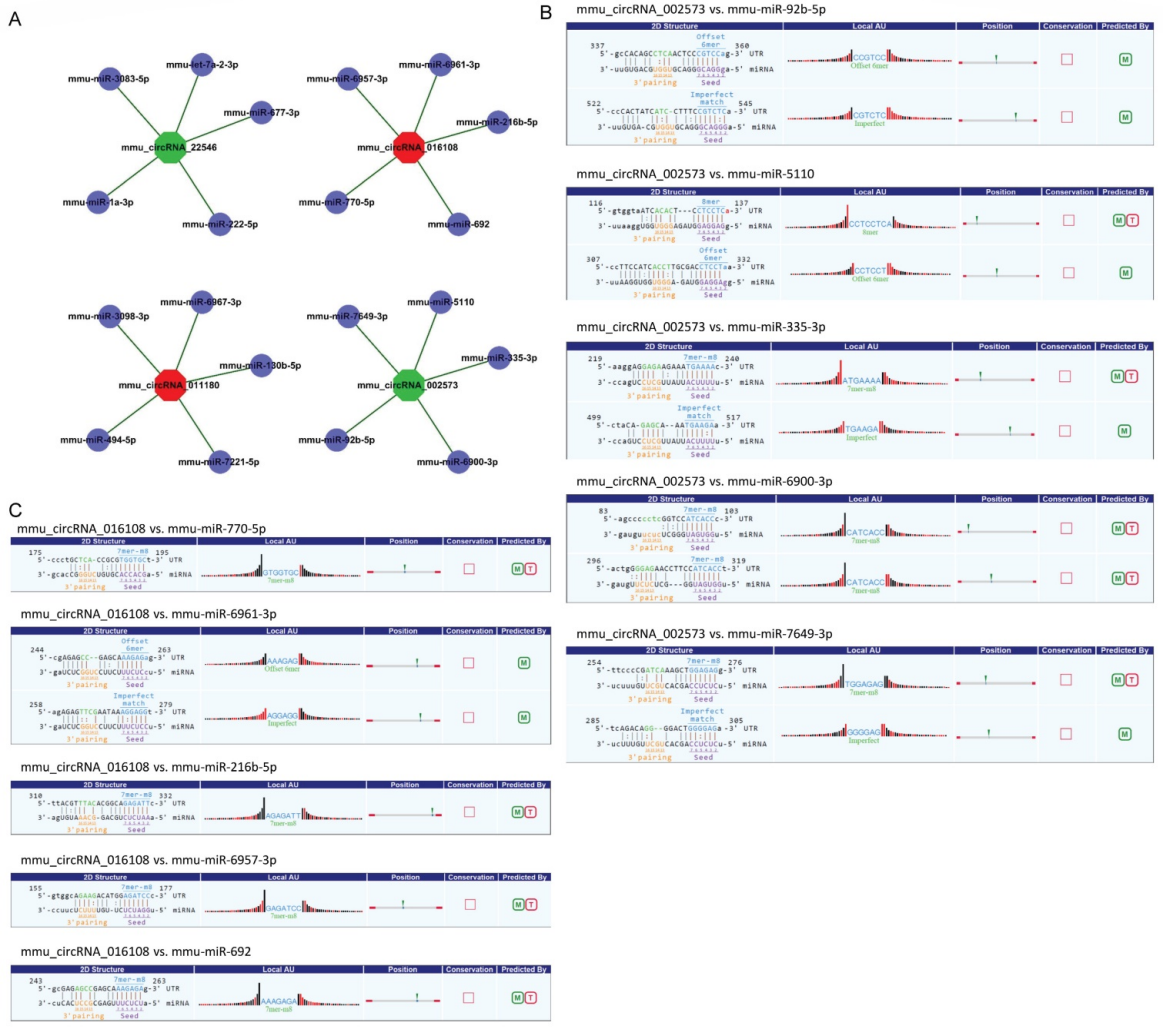
** Detailed annotation for circRNA-miRNA interaction. A.** Top 5 predicted targets of mmu_circRNA_011180, mmu_circRNA_016108, mmu_circRNA_22546 and mmu_circRNA_002573. **B.** Predicted interaction sites of mmu_circRNA_002573. **C.** Predicted interaction sites of mmu_circRNA_016108. M: be predicted by miRanda; T: be predicted by TargetScan.

CircRNA acted as microRNA sponge associating with related miRNAs, and together they made up circRNA-miRNA axis involving in disease pathogenesis. To determine the function of the validated circRNAs (mmu_circRNA_002573, mmu_circRNA_011180, mmu_circRNA_016108 and mmu_circRNA_22546), their target miRNAs were predicted by informatics analysis based on TargetScan as well as miRanda. The top 5 miRNAs related to each validated circRNA were shown in Fig. 3A, and the predicted interaction sites of mmu_circRNA_002573 and mmu_circRNA_016108 were also displayed in Fig. 3B-C.

### Competing endogenous RNA (ceRNA) regulatory networks with GO enrichment and KEGG pathway analyses

**Figure 4:**
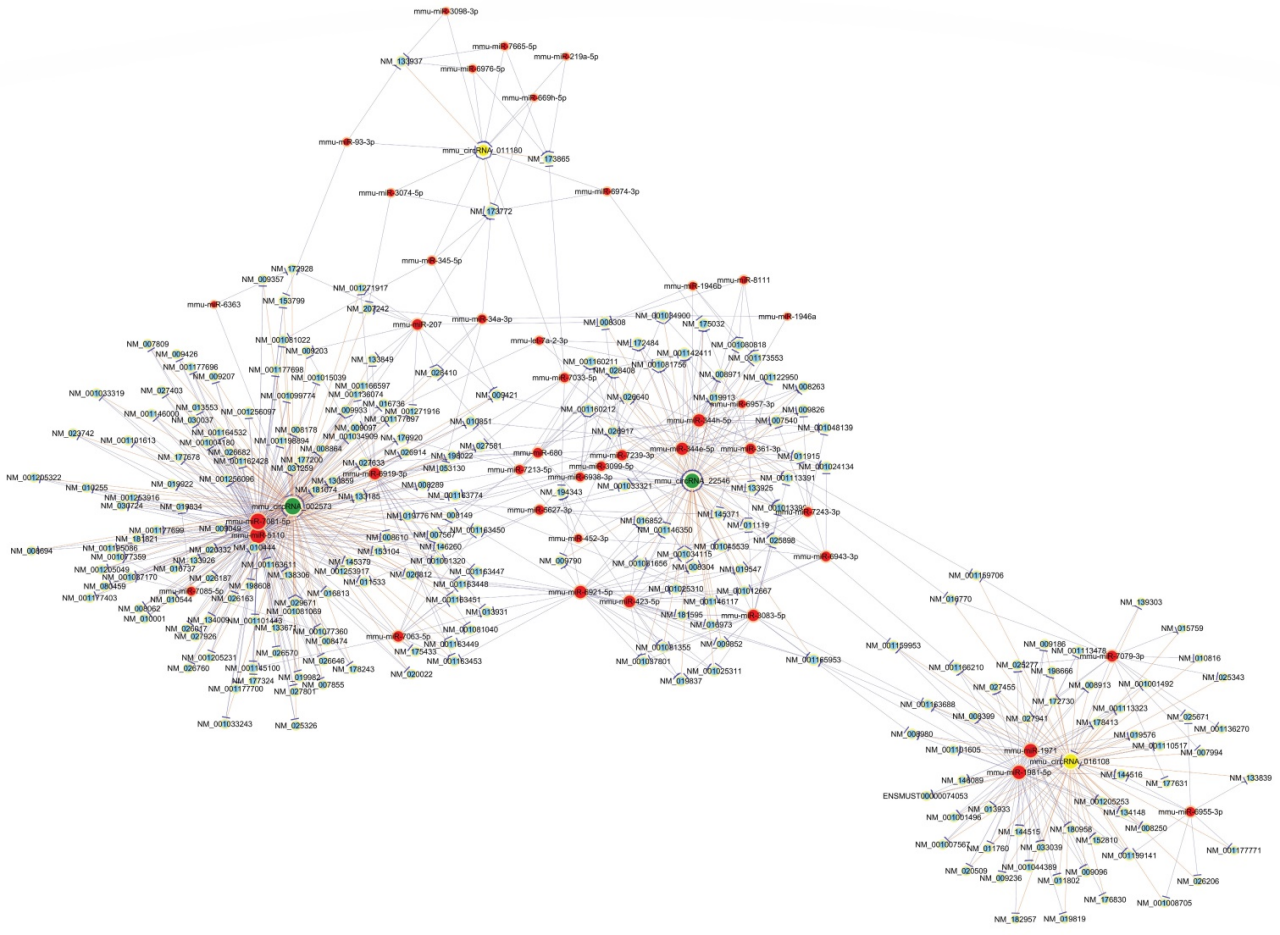
** The predicted circRNA‐miRNA‐mRNA networks.** The red color and light-blue color represent miRNA and mRNA, respectively. Yellow color and green color represent up-regulated and down-regulated circRNAs, respectively. Edges with T-shape arrow represent directed relationships, while edges without arrow represent undirected relationships.

To further elucidate the underlying mechanism of altered circRNAs we identified, construction of a circRNA-miRNA-mRNA regulatory network was generated by Cytoscape (Fig. 4). The network was constructed with 236 mRNAs, 4 circRNAs as decoys, and 42 predicted miRNAs in total. The network suggested that circRNAs could indirectly regulate miRNA target genes by competitively binding to miRNA through binding sites.

**Figure 5:**
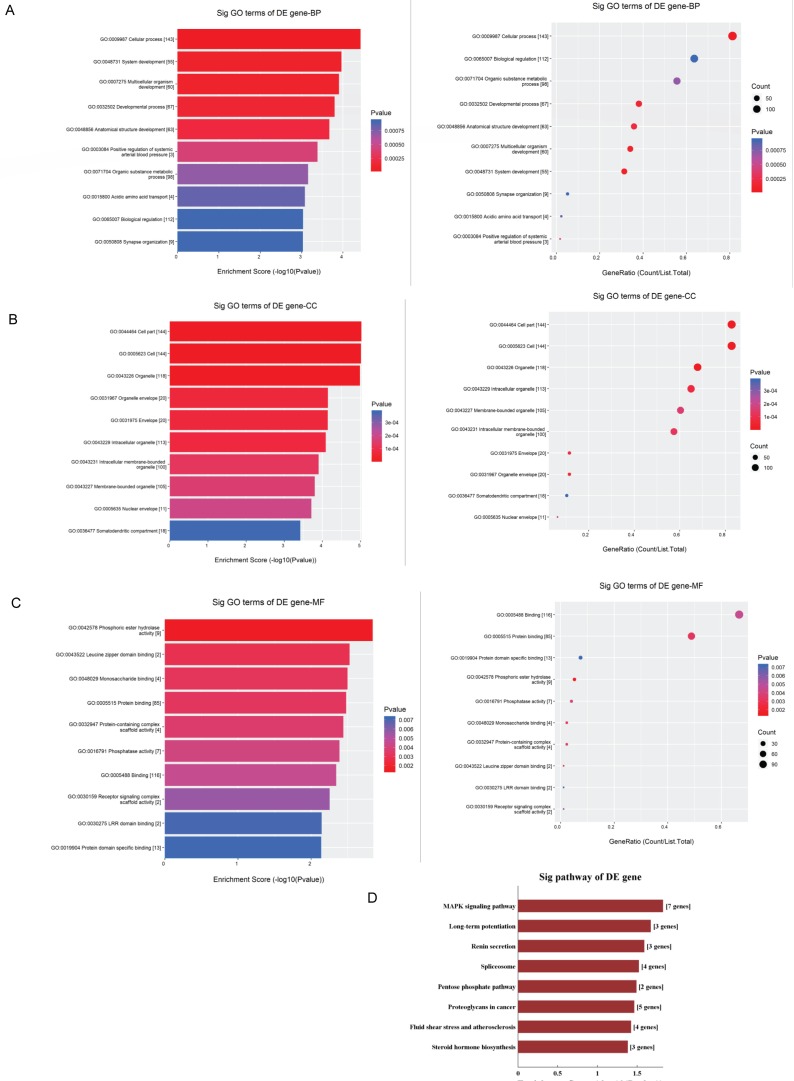
** GO and KEGG pathway analyses of validated circRNAs.** For **A-C,** parental genes according to the values in the enrichment score (left) and gene count (right) under the themes of biological processes, cellular components and molecular functions. The x‐ and y‐axis represent the top 10 significantly enriched terms. **A** represents biological processes. **B** represents cellular components. **C** represents molecular functions. **D.** The top enriched KEGG pathways of the significantly altered circRNA parental genes.

The GO analysis and KEGG pathway analysis were conducted to explore the biological function of the parental genes. The GO analysis revealed that the term with most genes and the term with the highest enrichment score were both cellular process (GO:0009987) for biological process (Fig.5A). For cellular component, the term “cell part” (GO: 0044464) was with most genes, and was the most significant enriched term as well (Fig.5B). Furthermore, binding (GO:0005488) was related with most genes and the term enriched most was phosphoric ester hydrolase activity (GO:0042578) (Fig.5C) for molecular function. Moreover, the enriched pathways analyzed by KEGG were MAPK signaling pathway, long-term potentiation, renin secretion, spliceosome, pentose phosphate, proteoglycans in cancer, fluid shear stress and atherosclerosis and steroid hormone biosynthesis (Fig.5D). These pathways were related to angiogenesis and endothelial cell motility.

## Discussion

Several studies have revealed roles of non-coding RNAs, particularly miRNA and lncRNA 13, 23. Some miRNAs, such as miR-23 and miR-27 served as promotors for neovascularization, while other miRNAs like miR-24 and miR-31 acted as inhibitors 23. Many miRNAs have been revealed to regulate crucial genes involved in the pathogenesis of ischemic retinopathy. For example, miRNA-126 maintained the integrity of the blood-retina barrier 24 and miR-29a inhibited retinal neovascularization to prevent ROP by down-regulating AGT 25. However, another spectrum of non-coding RNA, circRNA, was considered to be a by-product of error splicing and was overlooked for a long time. With closed-loop structures, circRNAs are more stable than linear RNAs and play essential roles in the pathogenesis of human diseases 26. A study reported that 529 circRNAs were abnormally expressed in diabetic retinas in human 15. Another study identified altered circRNAs in serum samples from patients with or without type 2 diabetes mellitus 14. Systemic dissection of circRNA profile in OIR model may provide clues to roles of circRNA in pathological neovascularization.

A few studies showed an intricate interplay between circRNAs and miRNAs. CircRNAs could competitively bind to miRNAs by miRNA-binding sites and the target genes were regulated as a result 27, 28. We examined circRNA-miRNA interactions and discovered that each selected circRNA, containing at least one miRNA binding sites, was able to interact with several miRNAs (Fig. 3). Our results suggested that circRNAs participated in OIR putatively through targeted miRNA and indirectly regulated gene expression. In addition, there is a subpopulation of circRNAs named exon-intron circRNAs (EIciRNAs), crosslink to RNA polymerase II, that remain in the nucleus and can regulate gene transcription directly *in cis* 29. However, the role of EIciRNAs in OIR models largely remains unclear.

The four validated circRNAs by qRT-PCR were selected to draw a whole picture of ceRNA regulatory networks. This circRNA-miRNA-mRNA network may provide clues to the regulatory pathways in OIR for the cascade-amplifying synergistic effects of circRNA-miRNA and miRNA-mRNA. Furthermore, GO enrichment analysis and KEGG pathway analysis were also performed to functionally annotate the predicted target genes. The ceRNA network revealed a novel interaction between the altered circRNAs and 236 mRNAs, and also showed that circRNA can regulate mRNAs through circRNA-miRNA binding. This network served as a shred of credible evidence that circRNA played a role in the pathogenesis of OIR by indirectly targeting certain mRNAs. GO analysis revealed that the most significant enriched term was cellular process in biological process, while cell part was the first highly ranked enriched term in cellular component and phosphoric ester hydrolase activity in molecular function.

The KEGG pathway analysis revealed several important pathways related to angiogenesis, including MAPK signaling pathway and renin-angiotensin system (RAS). A number of studies reported that angiogenesis can be restrained via downregulation of MAPK signaling pathway 30-32. Moreover, RAS was an essential growth factor which can stimulate angiogenesis in ischemia 33 and blockage of RAS could inhibit cancer angiogenesis 34. Altogether, GO and KEGG analysis suggested that mmu_circRNA_002573, mmu_circRNA_011180, mmu_circRNA_016108 and mmu_circRNA_22546, may take part in the process of neovascularization via different pathways.

Although we systemically profiled circRNA expression in retinas of OIR mice, limitations were existed in the study. For example, the circRNA expression profile should be interpreted with caution due to a limited number of samples. Secondly, false-negative data could be presented because the small quantity of circRNAs may below the detection threshold 35. Moreover, it is possible that the microarray-based screening might miss out some key circRNAs involved in retinal neovascularization, as microarray assays lack the sensitivity of advanced approaches such as next-generation sequencing. Apart from the limitations, there is a promising prospect for circRNAs in diagnosis and therapies since circRNAs are expressed widely and stably. For instance, circ_0005015 promoted retinal angiogenesis by regulating migration, proliferation as well as tube formation of the endothelial cells, and was able to act as potential biomarkers for diagnosis of diabetic retinopathy 15. A study suggested that exogenous introduction of circRNAs may stimulate the immune system for therapeutic purposes 36. In addition, circRNA HIPK3 promoted endothelial proliferation and vascular dysfunction by inhibiting miR-30a function 37. After intravitreal injection of AAV vectors encoding circRNA in mice, multiple retinal cell layers were observed with robust transgene expression, potentially providing novel therapeutic platforms of circRNAs 38.

In conclusion, our study demonstrated that the interactions of circRNAs and miRNAs may indirectly regulate gene expression and altered circRNA may play a role in retinal neovascularization. Further research on downstream molecular functions and their mechanisms may expose potential therapeutic targets for ischemia-induced retinal neovascularization.

## Figures and Tables

**Table 1:** Primers designed for qRT-PCR validation of selected circRNAs. Tm: temperature. bp: base pair

Gene name	Forward and reverse primer	Tm (℃)	Product length (bp)
GAPDH	F:5' CACTGAGCAAGAGAGGCCCTAT 3' R:5' GCAGCGAACTTTATTGATGGTATT 3'	60	144
mmu_circRNA_011180	F:5' CGTGACCACCCAGGAGACT 3' R :5' GCCATGTTGTCCACTTTCTC 3'	60	65
mmu_circRNA_016108	F:5' AGAGGAGAACGTGCAGATG 3' R :5' TCAGTGGGCAATGTTTCT 3'	60	87
mmu_circRNA_22546	F:5' CCAAGACCGATCACATCCC 3' R:5'GAAAAAGAGACAAGTTCTTCCTTGT 3'	60	78
mmu_circRNA_002573	F:5' GTGTTTACTGCCTTTGATGTGGTTT 3' R :5' CAAAATGCGTTTCCTGTGGC 3'	60	63

**Table 2:** Top 20 significantly up-regulated circRNAs by microarray analysis.

circRNA	P-value	FDR	FC (abs)	Regulation	chrom	strand	circRNA_type	GeneSymbol
mmu_circRNA_007438	0.014795	0.277447	2.446349	up	chr4	+	exonic	Gm20459
mmu_circRNA_31992	0.041523	0.316341	2.365270	up	chr18	+	sense overlapping	Zbtb7c
mmu_circRNA_29319	0.004326	0.254656	2.306132	up	chr16	-	exonic	Spidr
mmu_circRNA_36065	0.030444	0.305143	2.220547	up	chr3	-	exonic	6530403H02Rik
mmu_circRNA_004355	0.008633	0.267404	2.183836	up	chr7	-	sense overlapping	Serpinh1
mmu_circRNA_20332	0.035604	0.309920	2.139975	up	chr1	-	sense overlapping	Fn1
mmu_circRNA_32628	0.025983	0.300699	2.079943	up	chr19	-	exonic	Hpse2
mmu_circRNA_25277	0.034738	0.309017	2.078075	up	chr12	+	intronic	Psma3
mmu_circRNA_016108	0.026969	0.301802	2.039924	up	chr8	-	exonic	Klhl2
mmu_circRNA_011180	0.026366	0.300699	1.964496	up	chr8	-	exonic	Dlc1
mmu_circRNA_19534	0.023847	0.297676	1.962567	up	chr9	+	sense overlapping	Clasp2
mmu_circRNA_012082	0.044336	0.320710	1.919863	up	chr5	+	sense overlapping	Lrrc8d
mmu_circRNA_24234	0.033458	0.307110	1.917526	up	chr11	-	exonic	Kansl1
mmu_circRNA_45155	0.040614	0.315050	1.917268	up	chr9	+	exonic	Lrrc2
mmu_circRNA_19159	0.016640	0.281876	1.909133	up	chr16	-	sense overlapping	Spidr
mmu_circRNA_40299	0.018201	0.284126	1.891718	up	chr6	+	sense overlapping	Znrf2
mmu_circRNA_007784	0.020840	0.292410	1.886580	up	chr9	+	exonic	Elovl5
mmu_circRNA_26096	0.023428	0.297676	1.876620	up	chr13	-	exonic	AK045681
mmu_circRNA_43913	0.044269	0.320710	1.876312	up	chr9	+	exonic	Opcml
mmu_circRNA_36832	0.031896	0.305313	1.862768	up	chr4	-	exonic	Ptpn3

**Table 3:** Top 20 significantly down-regulated circRNAs by microarray analysis.

circRNA	P-value	FDR	FC (abs)	Regulation	chrom	strand	circRNA_type	GeneSymbol
mmu_circRNA_012434	0.011384	0.274663	3.135515	down	chr9	-	sense overlapping	Fat3
mmu_circRNA_37328	0.019337	0.287281	3.042786	down	chr4	+	exonic	Dhcr24
mmu_circRNA_19175	0.026023	0.300699	2.988649	down	chr17	+	intronic	Tulp4
mmu_circRNA_008009	0.023470	0.297676	2.958134	down	chr17	+	intronic	Tulp4
mmu_circRNA_41990	0.006321	0.260245	2.945088	down	chr7	+	exonic	Pgm2l1
mmu_circRNA_014393	0.014978	0.277447	2.911356	down	chr5	-	exonic	Zfp644
mmu_circRNA_39099	0.011233	0.274663	2.817654	down	chr5	-	intronic	Zfp644
mmu_circRNA_011865	0.028559	0.303367	2.801541	down	chr5	-	exonic	Zfp644
mmu_circRNA_39100	0.024888	0.298110	2.736680	down	chr5	-	intronic	Zfp644
mmu_circRNA_25930	0.043273	0.319272	2.616756	down	chr12	+	exonic	Rapgef5
mmu_circRNA_22265	0.013077	0.275894	2.613595	down	chr10	-	exonic	Trappc10
mmu_circRNA_30196	0.023838	0.297676	2.583449	down	chr17	+	intronic	Tulp4
mmu_circRNA_33363	0.006170	0.259846	2.549741	down	chr2	-	exonic	Strbp
mmu_circRNA_45453	0.018646	0.285878	2.541237	down	chrX	+	exonic	Ocrl
mmu_circRNA_19241	0.011866	0.274663	2.511977	down	chr2	-	sense overlapping	Strbp
mmu_circRNA_41991	0.018748	0.285878	2.501057	down	chr7	+	exonic	Pgm2l1
mmu_circRNA_014815	0.023080	0.297004	2.482177	down	chr4	-	exonic	Zfyve9
mmu_circRNA_006860	0.012424	0.274663	2.431044	down	chr5	-	exonic	Zfp644
mmu_circRNA_18971	0.004688	0.254656	2.424547	down	chr10	-	sense overlapping	L3mbtl3
mmu_circRNA_39101	0.024025	0.298110	2.383603	down	chr5	-	intronic	Zfp644

## Abbreviations

ceRNA: competing endogenous RNA
circRNA: circular RNA
EIciRNAs: exon-intron circRNAs
GO: Gene Ontology
KEGG: Kyoto Encyclopedia of Genes and Genomes
lncRNA: long non-coding RNA
miRNA: microRNA
OIR: oxygen-induced retinopathy
RAS: renin-angiotensin system
ROP: retinopathy of prematurity
qRT-PCR: quantitative real-time reverse transcription-polymerase chain reaction
VEGF: vascular endothelial growth factor
